# Comparative efficacy of interventions for improving quality of life, burden, distress, and symptoms of depression and anxiety in family and friend caregivers of people living with dementia

**DOI:** 10.1002/alz70858_102557

**Published:** 2025-12-25

**Authors:** Jennifer Watt, Zeest Kadri, Aryan Davoudi, Muhamad Ally, Mkeila Sowa, Areti Angeliki Veroniki, Andrea Tricco, Dallas Seitz, Zahinoor Ismail, Sharon Straus, Zahra Goodarzi

**Affiliations:** ^1^ University of Toronto, Toronto, ON, Canada; ^2^ Li Ka Shing Knowledge Institute, Toronto, ON, Canada; ^3^ Carleton University, Ottawa, ON, Canada; ^4^ Hotchkiss Brain Institute, University of Calgary, Calgary, AB, Canada; ^5^ University of Calgary, Calgary, AB, Canada

## Abstract

**Background:**

Family and friend caregivers of people with dementia (PwD) experience high distress, but have minimal access to support beyond education. Understanding interventions’ comparative efficacy at improving their mental health is critical.

**Method:**

We searched MEDLINE, Embase, CENTRAL, CINAHL, PsycINFO, and grey literature from inception until June 13, 2024, for randomized trials (RCTs) comparing any intervention to usual care or other interventions for improving quality of life, burden, distress, or depression or anxiety symptoms in PwD's family or friend caregivers. Independent reviewer pairs conducted study screening, data abstraction, and risk of bias appraisal. We derived standardized mean differences from random‐effects network meta‐analysis; back‐transformed mean differences (MD) on the World Health Organization Quality of Life Scale (psychological health), Zarit Burden Interview, Neuropsychiatric Inventory (distress), Center for Epidemiologic Studies Depression Scale, and Hospital Anxiety and Depression Scale (anxiety) to describe quality of life, burden, distress, and depression and anxiety symptom changes, respectively; and probabilities of exceeding each scale's minimum important difference (pMID).

**Result:**

We included 196 RCTs (83 interventions; 27,210 caregivers); 63.8% were at high risk of bias from missing data. Compared to education, psychotherapy (MD 17.4, 95% credible interval 3.5 to 31.7; pMID 95.1%) improved quality of life; education+training (‐6.4, ‐12.2 to ‐0.9; 57.8%) and caregiver/PwD exercise (‐13.1, ‐26.2 to ‐0.2; 85.9%) improved burden; mindfulness (‐8.3, ‐12.9 to ‐3.9; 97.6%) and case management+education (‐6.9, ‐13.0 to ‐0.3; 82.7%) improved distress; education+psychotherapy+support (‐35.3, ‐44.8 to ‐25.5; 100%), education+training (‐4.0, ‐6.2 to ‐1.7; 42.9%), education+psychotherapy (‐11.8, ‐21.0 to ‐2.6; 94.4%), mindfulness (‐5.1, ‐9.0 to ‐1.1; 66.4%), caregiver/PwD education (‐23.4, ‐35.9 to ‐9.9; 99.6%), respite care+education+support (‐23.1, ‐40.2 to ‐5.5; 98.2%), education+training+perspective‐taking (‐10.6, ‐20.7 to ‐0.6; 89.6%), and education+journaling+psychotherapy (‐7.0, ‐12.9 to ‐1.3; 83.5%) improved depressive symptoms; and education+training+psychotherapy (‐2.2, ‐3.7 to ‐0.8; 81.4%), education+support+training (‐3.1, ‐5.6 to ‐0.7; 89.0%), exercise+meditation (‐5.9, ‐9.2 to ‐2.7; 99.4%), cognitive behavioural therapy+support (‐15.4, ‐19.6 to ‐11.1; 100%), caregiver/PwD counselling (‐2.6, ‐5.2 to ‐0.1; 79.8%), and education+support+psychotherapy (‐7.2, ‐9.8 to ‐4.6; 100%) improved anxiety symptoms.

**Conclusion:**

Intervention combinations, with or without education, were more efficacious than education alone at improving mental health of PwD's family and friend caregivers.

